# Visual scanning behavior is related to recognition performance for own- and other-age faces

**DOI:** 10.3389/fpsyg.2015.01684

**Published:** 2015-11-03

**Authors:** Valentina Proietti, Viola Macchi Cassia, Francesca dell’Amore, Stefania Conte, Emanuela Bricolo

**Affiliations:** ^1^Department of Psychology, Brock UniversitySt. Catharines, ON, Canada; ^2^NeuroMI, Milan Center for NeuroscienceMilan, Italy; ^3^Department of Psychology, University of Milano-BicoccaMilan, Italy

**Keywords:** face age, age bias, eye movements, encoding, recognition, adult faces, infant faces

## Abstract

It is well-established that our recognition ability is enhanced for faces belonging to familiar categories, such as own-race faces and own-age faces. Recent evidence suggests that, for race, the recognition bias is also accompanied by different visual scanning strategies for own- compared to other-race faces. Here, we tested the hypothesis that these differences in visual scanning patterns extend also to the comparison between own and other-age faces and contribute to the own-age recognition advantage. Participants (young adults with limited experience with infants) were tested in an old/new recognition memory task where they encoded and subsequently recognized a series of adult and infant faces while their eye movements were recorded. Consistent with findings on the other-race bias, we found evidence of an own-age bias in recognition which was accompanied by differential scanning patterns, and consequently differential encoding strategies, for own-compared to other-age faces. Gaze patterns for own-age faces involved a more dynamic sampling of the internal features and longer viewing time on the eye region compared to the other regions of the face. This latter strategy was extensively employed during learning (vs. recognition) and was positively correlated to discriminability. These results suggest that deeply encoding the eye region is functional for recognition and that the own-age bias is evident not only in differential recognition performance, but also in the employment of different sampling strategies found to be effective for accurate recognition.

## Introduction

It is well-known that our ability to recognize faces varies depending on certain facial dimensions: individuals generally recognize human faces and faces from one’s own race more accurately and faster than other-species (see review in Dufour et al., 2006) and other-race faces (see review by Meissner and Brigham, 2001). Age as well is known to affect how faces are remembered. In a seminal study by Bäckman (1991), young adults recognized own-age faces more accurately than other-age faces regardless of whether the faces were familiar (famous) or unfamiliar. This original finding of an advantage in the processing of own-age compared to other-age faces (i.e., own-age bias, OAB) in young adults has been replicated in numerous studies investigating either identity recognition (in eyewitness paradigms or old/new recognition tasks) or identity matching (in delayed match-to-sample tasks) when performance for young adult (i.e., own-age) faces was compared to that for older adult faces (e.g., Anastasi and Rhodes, 2006; Wiese et al., 2008; He et al., 2011) child faces (Anastasi and Rhodes, 2005; Kuefner et al., 2008; Harrison and Hole, 2009; Hills and Lewis, 2011) or infant faces (Kuefner et al., 2008; Macchi Cassia et al., 2009a,b; Yovel et al., 2012).

For all these dimensions, the faces that are more readily recognized—that is, human faces, own-race faces and own-age faces—when compared with their within-category counterparts—that is, other-species faces, other-race faces and other-age faces—are those with which participants have accumulated abundant experience. Superior recognition of faces from over-experienced categories has been attributed to perceptual expertise as well as to social cognitive factors. According to perceptual expertise accounts, extensive experience with faces from a given category (e.g., own-race) results in exquisite sensitivity to differences among faces in, for example, the shape and spacing of facial features (e.g., Rhodes et al., 2009; Tanaka and Pierce, 2009; Mondloch et al., 2010). According to social cognitive accounts, adults encode faces of in-group members at the individual level whereas they encode faces of out-group members at the categorical level (Levin, 2000; Sporer, 2001; Ge et al., 2009). Recent proposals have argued for an integrative framework in which social cognition and perceptual expertise interact in determining an individual’s sensitivity to individuating facial characteristics (Sporer, 2001; Young et al., 2012).

Indeed, there is ample evidence that adults process faces from different races differently, both in terms of the underlying neural mechanisms and the associated visual processing strategies. For example, electrophysiological studies have found that the face-sensitive N170 is of larger amplitude in response to upright other-race faces compared to upright own-race faces and face inversion affects this component more for the latter than the former types of faces. These results suggest that although configural/holisitic information is extracted from faces of both racial groups, upright other-race faces require increased processing demands (e.g., Caharel et al., 2011; Montalan et al., 2013). Although results are not always consistent, several behavioral studies have suggested that both configural/holistic information (e.g., Tanaka et al., 2004; Michel et al., 2006) and featural cues (e.g., Hayward et al., 2008; Mondloch et al., 2010) are extracted more effectively from own-race faces than other-race faces.

More recently, the question of whether, and to what extent, the own-race bias in face memory is related to perceptual processing differences has been productively addressed using eye-tracking methodologies, which provide a direct measure of visual scanning behavior through on-line recording of visual fixations on various portions of the face with high temporal and spatial resolution. When viewing faces, adults are found to spend more time fixating the internal features, e.g., the eyes, nose and mouth (e.g., Janik et al., 1978; Walker-Smith et al., 2013), and this scanning strategy is related to subsequent recognition (e.g., Henderson et al., 2005). Given that eye movements are important for face memory, several studies have explored whether recognition deficits observed for faces belonging to less familiar race groups can be related to non-optimal exploration of these faces during encoding and/or recognition. Conflicting findings have been obtained in the investigation of this hypothesis. Some recent studies have shown how culture affects the way people view faces: Western observers normally tend to look longer to the eye region (reflecting the use of analytic perceptual strategies), whereas East Asians tend to focus more on the nose region (possibly reflecting the use more holistic perceptual strategies; Blais et al., 2008; Caldara et al., 2010; Fu et al., 2012; Hills and Pake, 2013). While some studies found that these cross-cultural variations in scanning strategies do not differ for own- compared to other-race faces (Blais et al., 2008; Caldara et al., 2010; Hills and Pake, 2013), other studies showed that these variations are modulated by face race (East Asian participants: Fu et al., 2012; Hu et al., 2014; Western participants: Goldinger et al., 2009; Wu et al., 2012; McDonnell et al., 2014). Western participants were found to make more fixations on the eye region of same-race faces compared to other-race faces, and to fixate longer the nose and mouth region of Asian compared to Caucasian faces (e.g., Goldinger et al., 2009); they are also reported to make a larger number of shorter fixations while exploring own-race compared to other-race faces, suggesting the use of more active scanning strategies for the former than the latter (e.g., Wu et al., 2012). The same pattern of scanning behavior is observed during recognition, as the eyes of same-race faces are sampled more often than those of other-race faces, whereas the opposite occurs for the mouth (Nakabayashi et al., 2012).

Unlike the own-race bias, investigations of how faces of different ages are perceptually encoded and processed are limited. The behavioral own-age recognition advantage is mirrored in young adults by ERP responses, which show higher degree of specialization for own-age faces (i.e., young faces) compared to other-age faces (i.e., older faces; larger N170, VPP, frontocentral P200 for older compared to young faces; larger occipital P200 for young compared to older faces; Wiese et al., 2008, 2012; Ebner et al., 2011). However, evidence of perceptual processing differences between adult faces and faces belonging to other-age groups comes mainly from studies comparing the disrupting effects produced on the discrimination of those faces by stimulus manipulations that are known to hinder configural and/or holistic processing, like the face inversion effect (e.g., Kuefner et al., 2008) and the composite-face effect (e.g., de Heering and Rossion, 2008). These studies have shown that adults rely more heavily on expert configural/holistic strategies when processing own-age faces compared with elderly adult faces (Proietti et al., 2013; Wiese et al., 2013), child faces (de Heering and Rossion, 2008; Kuefner et al., 2008, 2010), and infant faces (Macchi Cassia et al., 2009a).

Critically, although this evidence clearly supports the hypothesis of a perceptual processing advantage for younger adult faces compared to a wide range of other-age face types, investigations of how individuals visually scan own- and other-age faces, and how differences in scanning behavior may relate to different recognition performance are quite limited. To the best of our knowledge, only three studies have addressed this question by recording young adult participants’ eye movements through eye-tracking methodologies, and all focused on the comparison between young adult (i.e., own-age) and elderly adult faces (Firestone et al., 2007; He et al., 2011; Short et al., 2014). Results converge in showing that young adults look longer at own-age faces compared to older adult faces, both when faces are presented in isolation (Firestone et al., 2007; He et al., 2011) and when they are embedded in naturalistic scenes and the two face ages directly compete for attention (Short et al., 2014).

Among these studies, though, only Firestone et al. (2007) actually investigated whether the distribution of eye movements across various facial regions differed for young and older adult faces, as Short et al. (2014) considered each face as a whole region of interest, and He et al. (2011) only divided each face into lower and upper half and found no difference in distribution of looking time across the two regions between young and older adult faces. Firestone et al.’s (2007) results confirmed the general tendency of Caucasian observers to look longer at the eyes region, followed by the nose and the mouth region. However, although young (i.e., own-age) faces received more transitions between facial regions compared to older adult faces, they also received a decrease in sampling of the eyes, and an increase in sampling of the nose and mouth compared to older faces. Moreover, the authors found that, irrespectively of face age, increased looking time on the nose region was associated to successful subsequent recognition. The authors concluded that patterns of eye scanning during the encoding of unfamiliar faces are critically related to recognition. However, the finding that looking at the nose, rather than at the eye region, mediated correct identification is at odds with demonstrations that longer looking at the upper facial regions (i.e., hair, eyes) results in more accurate recognition of own-race faces (McDonnell et al., 2014).

The aim of the present study was to extend available evidence on the relationship between visual scanning behavior and recognition performance for own- and other-age faces by comparing eye movement scanning patterns exhibited by young adult participants while encoding and recognizing adult and infant faces within the context of an old/new recognition memory task. Infant faces were chosen because, given that newborns are very infrequently present in an adult’s typical everyday environment, the amount of individual’s exposure to this specific face category is very limited and can be estimated rather well. The influence of experience with infant faces was controlled in the study by selecting participants for having null or limited direct contact with infants (i.e., infant novices), according to the same criteria used in previous studies comparing discrimination and processing abilities for adult and infant faces (Kuefner et al., 2008; Macchi Cassia et al., 2009a,b; see also Yovel et al., 2012). In these studies, infant novices showed better discrimination for young adult faces compared to infant faces in a delayed two-alternative forced choice matching-to-sample task, in which they were asked to match a briefly presented target face to two simultaneously presented test faces appearing after a short delay. Critically, adult participants also showed an inversion effect that was selective for young adult faces. Because it is well-established that at least a portion of the inversion effect is related to configural processing of upright faces (Mondloch et al., 2002), the authors interpreted the complete absence of an inversion effect for infant faces as evidence that configural processing was not engaged to any extent for the recognition of these faces.

In light of this evidence, the present study had three main goals: (1) to extend available evidence of a recognition bias for adult over infant faces using an old/new recognition memory task; (2) to investigate whether adults show differences in gaze patterns while encoding and/or recognizing adult and infant faces; (3) to test whether these differences in gaze patterns are related to recognition performance. Based on the overarching hypothesis that face recognition varies as a function of expertise with different face categories (i.e., own- vs. other-age faces) and that such improvement may be explained by differential visual encoding strategies, we predicted that: (1) participants would show an own-age recognition advantage as indicated by higher recognition accuracy and/or lower response times (RTs) for adult compared to infant faces; (2) looking behavior (looking time and the dynamicity of visual exploration) would differ for adult and infant faces; (3) recognition performance would vary as a function of looking behavior, possibly with longer fixations on the upper regions of the face being linked to more efficient subsequent recognition.

## Materials and Methods

### Participants

Participants were 27 female university students aged from 19 to 29 years (*M* = 23.89 years, *SD* = 2.05). They were asked to participate if they had no offspring and had not acquired extensive experience with infants (i.e., 2 years or younger). To this end, potential participants were screened prior to testing via a questionnaire that included specific inquiries aimed at assessing whether, in the past 5 years, they had had nieces or nephews, contact with infants of friends or acquaintances, and/or a job that put them in contact with infants. Inclusion criteria were identical to those of earlier studies investigating the own-age bias in participants with little or no experience with infants (Macchi Cassia et al., 2009a,b; i.e., less than 520 h of experience per year in the past 5 years). Participants included in the sample had acquired an average of 91.48 h (*SD* = 114.62, range = 0–520) of experience per year over the past 5 years. All participants were Italian and right-handed, and they all had normal or corrected-to-normal vision. All procedures used in the current study complied with the Ethics Standards outlined by the Declaration of Helsinki (BMJ 1991; 302: 1194) and were approved by the Ethics Committee of the University of Milano-Bicocca. All participants signed an informed consent before testing and received formation credits for their participation.

### Stimuli

**FIGURE 1:**
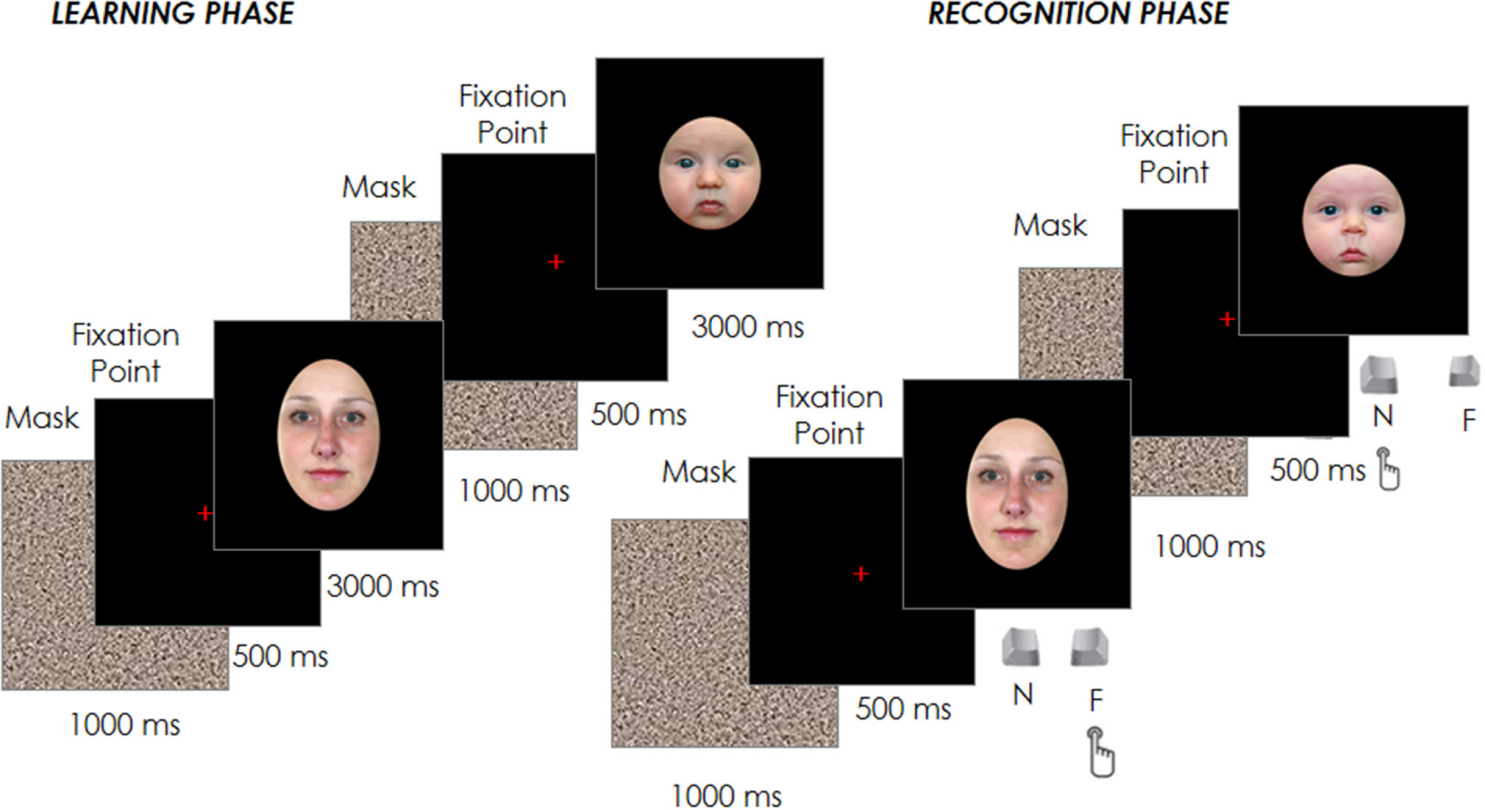
**Schematic representation of the experimental design**.

Twenty-four color photos of female adult faces and 24 photos of infant (aged 3–5 months) faces were used as stimuli. Faces were all Caucasian, frontal, and with neutral expression; an oval-shaped occluder was placed on each face to conceal background information (e.g., hair and ears*;* **Figure 1**). The hue and brightness of the color face images (resolution 72 dpi) were leveled out and were all normalized to be of the same width (306 pixel, 7.2 cm, 6.3° of visual angle). The height of the stimuli and consequently of the occluder differed between the two types of faces in order to maintain ecological validity (adult faces = 10.6 cm, 9.3° of visual angle; infant faces = 7.94 cm, 7° of visual angle). Faces in each age group were normalized to be the same shape and size. Moreover the eyes, nose, and mouth position were normalized to the locations of the eyes, nose, and mouth of the average image computed on the 24 stimuli, so that the major features of all face stimuli were located in the same face regions.

### Apparatus

All faces appeared on a light gray background at the center of the 19 inches Samsung SyncMaster 1200 NF screen, with a resolution of 1024 × 768 pixels. Stimulus presentation and response collection were controlled by the E-prime 2.0 software. Participants’ eye movements were recorded using an Applied Science Laboratories’ (ASL) Model 504 Eye Tracker 6 system. Participants had their head on a chin-rest and sat about 65 cm from the eye tracker camera located at the base of the presentation screen, which measured participants’ eye movements at a sampling rate of 50 Hz.

### Procedure

Participants were tested in an old/new face recognition task while their eye movements were recorded. A manual calibration of gaze position was conducted at the beginning of the testing session, and repeated at the beginning of each experimental block, using a nine-point fixation procedure. The calibration was validated and repeated when necessary until the optimal calibration criterion was reached.

Each trial started with a fixation cross at the center of the screen, which participants had to fixate for 500 ms in order for the target face to appear for 3000 ms. Participants were instructed to inspect carefully and memorize a sequence of 12 adult and 12 infant faces presented in random order in the center of the screen. Each face was spaced out by a 1000 ms gray noise mask to reduce a possible retinal permanence effect, followed by the 500 ms fixation point (see **Figure 1**).

After the 24 trials of the learning phase, participants performed a filler task used to create a temporal gap between the learning and the recognition phase and to reduce any potential recency effects. In brief, this filler involved an object search in which participants were asked to identify a specific shape (e.g., a square) among other distractor shapes (e.g., triangles). Once identified, a new trial would begin and this process would repeat until 3 min had elapsed. Participants’ eye movements during this filling task were not recorded and their performance was not analyzed. Immediately afterward, the test phase began with the presentation of the 24 familiar faces previously seen in the learning phase plus other new 24 faces (12 adult and 12 infant) randomly intermixed with the formers. Each trial started with a fixation cross at the center of the screen, which participants had to fixate for 500 ms in order to have the target face appear. The face remained on the screen until the participant had classified the face as familiar (already seen in the learning phase) or novel by pressing one of two joystick buttons (**Figure 1**). The face images presented in both the learning and test phases were counter-balanced between participants, as was the response associated with the joystick buttons.

## Results

### Behavioral Performance

Three behavioral performance measures were computed for each participant separately for responses to adult and infant faces (sensitivity index -d’-, response bias -c- and mean correct RTs -RTs-) and analyzed to test our first prediction that participants would show an own-age recognition advantage as revealed by higher recognition accuracy and/or lower RTs for adult compared to infant faces. **Table 1** shows the mean and standard error of the mean (SE) for each measure. To assess the own-age bias on recognition data, we conducted paired sample *t*-tests to compare each measure of performance between adult and infant faces (**Table 1**). Participants performed more accurately in the recognition of adult compared to infant faces, as indicated by the significant difference emerged in the sensitivity index (d’), *t*(26) = 2.226, *p* = 0.035 (i.e., higher d’ for adult faces compared to infant faces). The comparisons for response bias and mean RTs did not reach statistical significance (c: *p* = 0.074; mean RTs: *p* = 0.094), although the pattern for mean RTs was in the expected direction. It is not unusual to obtain a recognition bias on some measures but not others in similar tasks (Meissner and Brigham, 2001; McDonnell et al., 2014), therefore our data reflected the presence of an OAB.

**Table 1:** Behavioral performance measures: means and SE of sensitivity index (d’), response bias (c), mean correct response times (RTs) in milliseconds for adult and infant faces.

	Adult faces		Infant faces		Comparison between adult and infant faces	
	*M*	(*SE*)	*M*	(*SE*)	T	*p*-value
d’	1.48	(0.11)	1.24	(0.10)	2.226	**0.035**
c	0.23	(0.07)	0.06	(0.07)	1.861	0.074
RT	1664.86	(102.44)	1806.02	(149.15)	-1.738	0.094

*p-values in bold are significant (p < 0.05).*

### Eye Movements

**FIGURE 2:**
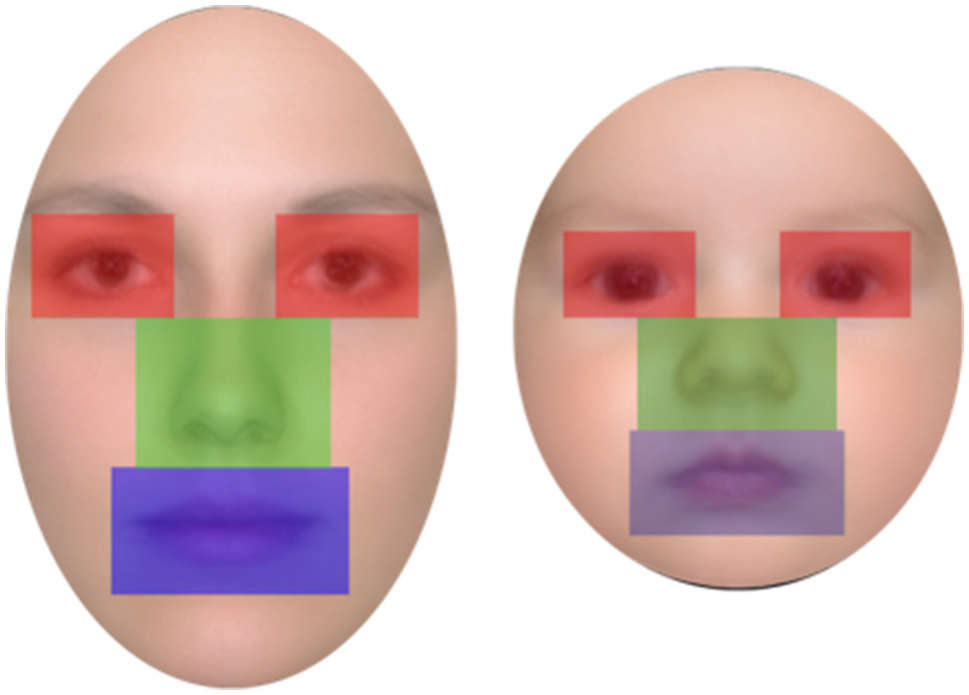
**Example of areas of interest (AOI) plots for adult faces (left) and infant faces (right)**.

Participants’ eye movement scanning behavior was analyzed for both the learning and the recognition phases in order to test our second prediction that looking behavior would differ for adult and infant faces. Three areas of interest (AOIs) were defined for each face of the two age groups: the eyes (right and left combined), the nose, and the mouth (see **Figure 2**). The three AOIs were equal in size and, together, covered 36 % of the total area of the face (each AOI covered 12% of the face). Thus, the proportion of the face captured by the AOIs was held constant for adult and for infant faces (see **Figure 2**).

Two measures were derived from eye movement data: percentage of total viewing time on each AOI and the number of visits per unit time (second) across all AOIs. The first was created to provide a measure of the relative amount of sampling of each facial feature, while the second was created to index the dynamicity of visual processing across the whole face. The percentage of total viewing time was calculated for each trial by dividing the total fixation time on each AOI by the total fixation time on the whole face, and by multiplying the result by 100. Percentages, rather than raw viewing time, were used in order to directly compare viewing time across the learning and recognition phase, which differed for trial duration (learning: 3 s, recognition: until response, *M* = 1735.44 ms, *SE* = 127.47). It should be noted that the total fixation time on the three AOIs (eyes, nose, and mouth) did not add to 100% of the on-face fixation time because some fixations may have fallen outside the AOIs but still within the face area. Number of visits per second was calculated for each trial by dividing the total number of visits (number of times the gaze entered a specific AOI in a given trial) received by each AOI by trial duration, in seconds, which was fixed to 3 s for learning trials, and variable until response for recognition trials. For this measure the left and right eyes were considered as separate AOIs.

Different sets of analyses were performed for each eye movement measure. A first set included eye movement data from all the trials. Furthermore, to test our third prediction that recognition performance would vary as a function of looking behavior, a second and a third set of analyses were performed separately for trials that triggered a correct response during the recognition phase and those that were incorrectly recognized. Separate analyses were performed for the two response measures because, while all participants made at least one correct response on both adult and infant trials, two participants did not have any incorrect response in at least one of the two conditions. For all sets of analyses, analyses of variance (ANOVAs) were conducted on each of the eye movement measures using the factors face age (adult, infant), phase (learning, recognition), and, for total viewing time, AOI (eyes, nose, mouth). All comparisons were Bonferroni corrected.

#### Analyses on All Trials

##### Percentage of total viewing time

**FIGURE 3:**
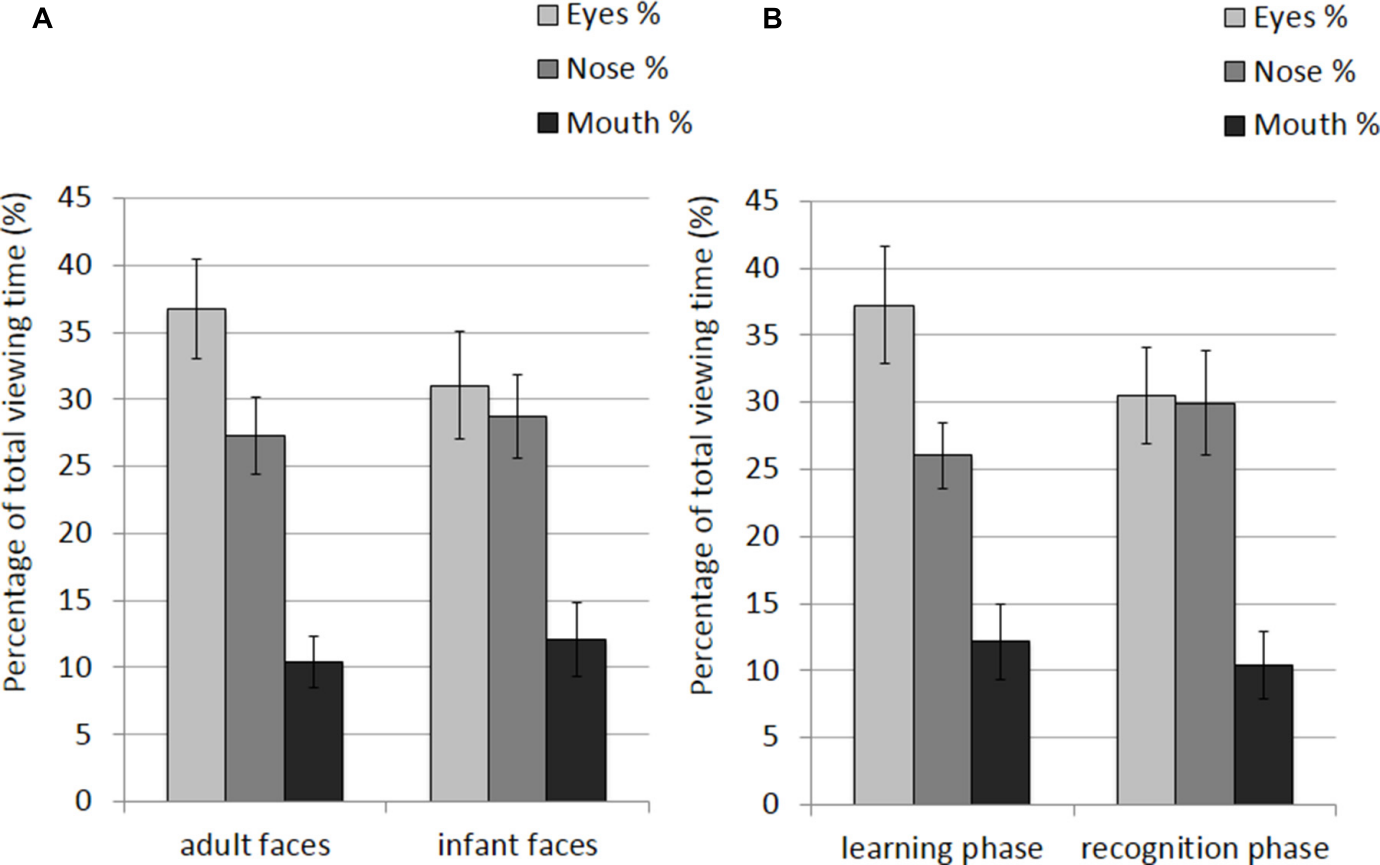
**Percentage of total viewing time recorded on all trials plotted as a function of AOIs (eyes, nose, and mouth) for: (A) adult and infant faces; (B) the learning and recognition phase.** Error bars represent the standard error of the means.

The mean and SE of the percentage of total viewing time on each AOI for the adult and infant faces during learning and recognition are shown in **Table 2**. The 2 × 2 × 3 ANOVA showed a significant main effect of AOI, *F*(2,52) = 16.582, *p* < 0.001, η^2^ = 0.389. Bonferroni-corrected, multiple-comparison tests revealed an overall smaller percentage of viewing time on the mouth region (*M* = 11.26%, *SE* = 1.92%) compared to both the nose region (*M* = 27.99%, *SE* = 2.39%), *p* < 0.001, and the eye region (*M* = 33.87%, *SE* = 3.16%), *p* < 0.001. No differences emerged between the eye and the nose regions (*p* > 0.74). The AOI main effect was qualified by two significant interactions between AOI and face age, *F*(2,52) = 6.999, *p* = 0.002, η^2^ = 0.212, and AOI and phase*, F*(2,52) = 3.958, *p* = 0.025, η^2^ = 0.132 (see **Figure 3**). *Post hoc* pairwise *t*-tests showed that the only significant difference between adult and infant faces across the two phases concerned the percentage of viewing time on the eyes, which was higher for adult faces (*M* = 36.71%, *SE* = 3.13%) than for infant faces (*M* = 31.03%, *SE* = 3.36%), *t*(26) = 3.775, *p* = 0.003. The mouth region was the least fixated area of the three AOIs for both adult and infant faces, *p*s < 0.01. *Post hoc t*-tests also showed that participants looked significantly longer at the eye AOI during learning (*M* = 37.26%, *SE* = 3.69%) compared to recognition (*M* = 30.48%, *SE* = 3.01%), *t*(26) = 2.878, *p* = 0.024. No other difference was found to be significant, *p*s > 0.14. Also, in both the learning and recognition phase, the mouth region was the least fixated area of the three AOIs, *p*s < 0.007.

**Table 2:** Mean and standard error of the percentage of total looking time on each of the three AOI (eyes, nose, mouth) on the adult and infant face during learning and recognition phase.

	Learning				Recognition			
AOI	Adult faces		Infant faces		Adult faces		Infant faces	
	*M*	(*SE*)	*M*	(*SE*)	*M*	(*SE*)	*M*	(*SE*)
**All trials**								
Eyes	40.74	(4.03)	33.78	(3.53)	32.68	(3.15)	28.28	(3.45)
Nose	25.28	(2.11)	26.79	(2.32)	29.19	(3.24)	30.68	(3.77)
Mouth	11.12	(1.86)	13.16	(3.03)	9.69	(1.82)	11.07	(2.41)

*Reported data refer to all the trials (correct and incorrect).*

##### Number of visits per second

**FIGURE 4:**
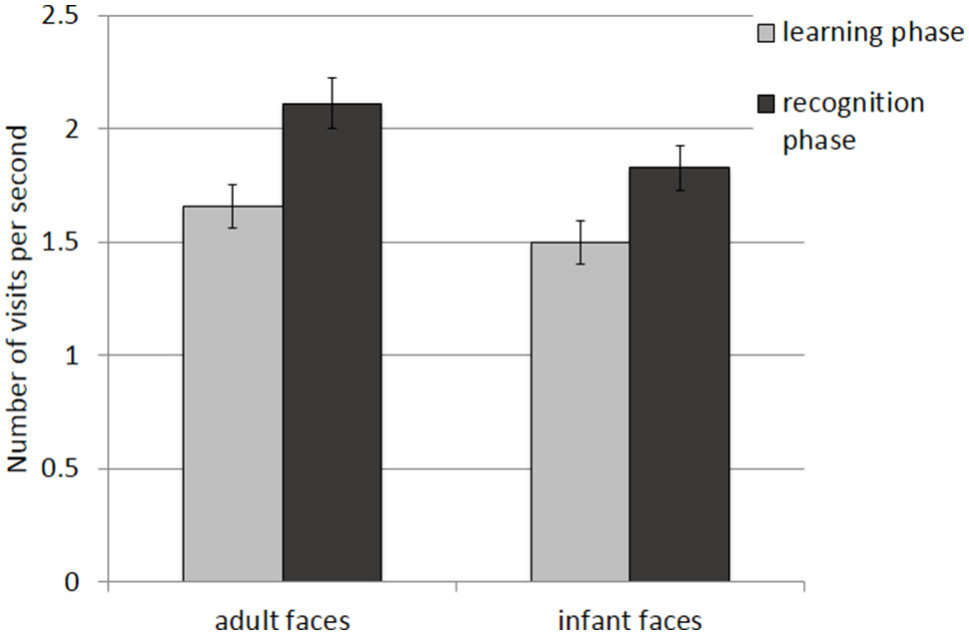
**Number of visits per second recorded on all trials during the learning and recognition phase for adult and infant faces.** Error bars represent the standard error of the means.

The mean and SE of the number of visits for the adult and infant faces during learning and recognition are shown in **Table 3**. The 2 × 2 ANOVA with face age and phase as within-subjects factors revealed main effects of both face age*, F*(1,26) = 33.370, *p* < 0.001, η^2^ = 0.562, and phase, *F*(1,26) = 27.916, *p* < 0.001, η^2^ = 0.518, indicating that participants made more visits per second while encoding adult faces (*M* = 1.89, *SE* = 0.09) compared to infant faces (*M* = 1.66, *SE* = 0.09) and they made more visits per second when recognizing faces (*M* = 1.97, *SE* = 0.10) than during learning (*M* = 1.58, *SE* = 0.09; see **Figure 4**).

**Table 3:** The mean and standard error of the number of visit per second for the adult and infant face conditions separately for the learning and recognition phases.

	Learning				Recognition			
	Adult faces		Infant faces		Adult faces		Infant faces	
	*M*	(*SE*)	*M*	(*SE*)	*M*	(*SE*)	*M*	(*SE*)
All trials	1.66	(0.09)	1.49	(0.09)	2.11	(0.11)	1.83	(0.10)
Correct trial	1.65	(0.09)	1.49	(0.09)	2.14	(0.11)	1.85	(0.10)
Incorrect trials	1.58	(0.11)	1.37	(0.11)	1.99	(0.11)	1.74	(0.10)

*Mean and SE values refer to all the trials (first line), correct trials (second line) and incorrect trials (third line).*

#### Analyses on Correct Trials

##### Percentage of total viewing time

**FIGURE 5:**
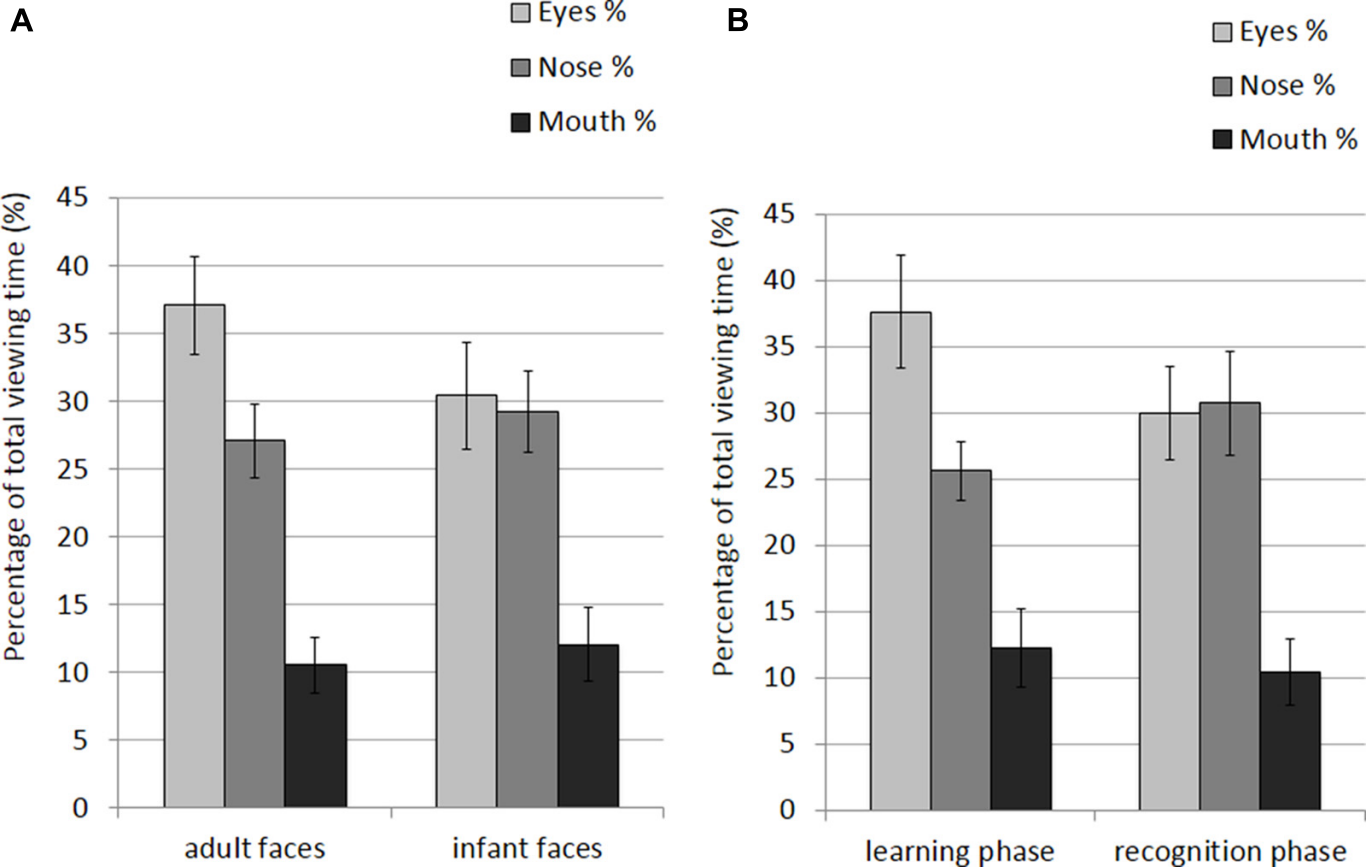
**Percentage of total viewing time recorded on correct trials plotted as a function of AOIs (eyes, nose and mouth) for: (A) adult and infant faces; (B) the learning and recognition phase.** Error bars represent the standard error of the means.

The mean and SE of the percentage of total viewing time on each AOI for the adult and infant faces during learning and recognition are shown in **Table 4**. The 2 × 2 × 3 ANOVA revealed main effects of face age, *F*(1,26) = 5.07, *p* = 0.033, η^2^ = 0.163, and AOI, *F*(1,26) = 17.366, *p* < 0.001, η^2^ = 0.400. Participants spent longer looking at the three AOIs for adult faces (*M* = 24.92%, *SE* = 1.0%) compared to infant faces (*M* = 23.91%, *SE* = 1.02%). Bonferroni-corrected, multiple-comparison tests revealed an overall smaller percentage of viewing time on the mouth region (*M* = 11.32%, *SE* = 1.97%) compared to both the nose region (*M* = 28.16%, *SE* = 2.29%), *p* < 0.001, and the eye region (*M* = 33.77%, *SE* = 3.08%), *p* < 0.001. Viewing time did not differ between the eye and the nose regions (*p* > 0.75). The AOI main effect was qualified by two significant two-way interactions with the factor face age, *F*(1,26) = 10.330, *p* < 0.001, η^2^ = 0.284, and phase, *F*(1,26) = 5.462, *p* = 0.007, η^2^ = 0.174 (see **Figure 5**). The percentage of time that participants spent viewing the eye region was higher for adult faces (*M* = 37.09%, *SE* = 3.02%) compared to infant faces (*M* = 30.46%, *SE* = 3.31%), *t*(26) = 4.455, *p* < 0.001, whereas there was no significant difference between the two face ages on viewing time on the nose, *p* = 0.155, and mouth, *p* = 0.137. For both face ages, the mouth AOI was the least fixated region, *p*s < 0.01. The AOI × phase interaction was due to the fact that participants spent more time viewing the eye region during the learning phase (*M* = 37.61, *SE* = 3.601%) compared to the recognition phase (*M* = 29.93%, *SE* = 2.97%), *t*(26) = 3.266, *p* = 0.021. Furthermore, in the recognition phase both the eye and the nose regions were viewed more than the mouth, *p*s < 0.001.

**Table 4:** The mean and standard error of the percentage of total looking time on each of the three AOI (eyes, nose, mouth) on the adult and infant faces separately for the learning and recognition phases.

	Learning				Recognition			
AOI	Adult faces		Infant faces		Adult faces		Infant faces	
	*M*	(*SE*)	*M*	(*SE*)	*M*	(*SE*)	*M*	(*SE*)
**Correct trials**								
Eyes	41.98	(3.97)	33.24	(3.46)	32.19	(3.03)	27.67	(3.50)
Nose	23.99	(1.76)	27.18	(2.32)	30.23	(3.29)	31.24	(3.68)
Mouth	11.46	(2.14)	13.00	(2.90)	9.69	(1.82)	11.12	(3.45)
**Incorrect trials**								
Eyes	35.48	(4.08)	31.63	(4.26)	30.15	(3.49)	27.29	(3.39)
Nose	27.68	(2.88)	24.99	(3.12)	26.42	(3.52)	29.92	(4.63)
Mouth	9.42	(1.79)	12.67	(3.67)	9.73	(2.05)	11.58	(2.62)

*Data are presented separately for correct and incorrect trials.*

##### Number of visits per second

**FIGURE 6:**
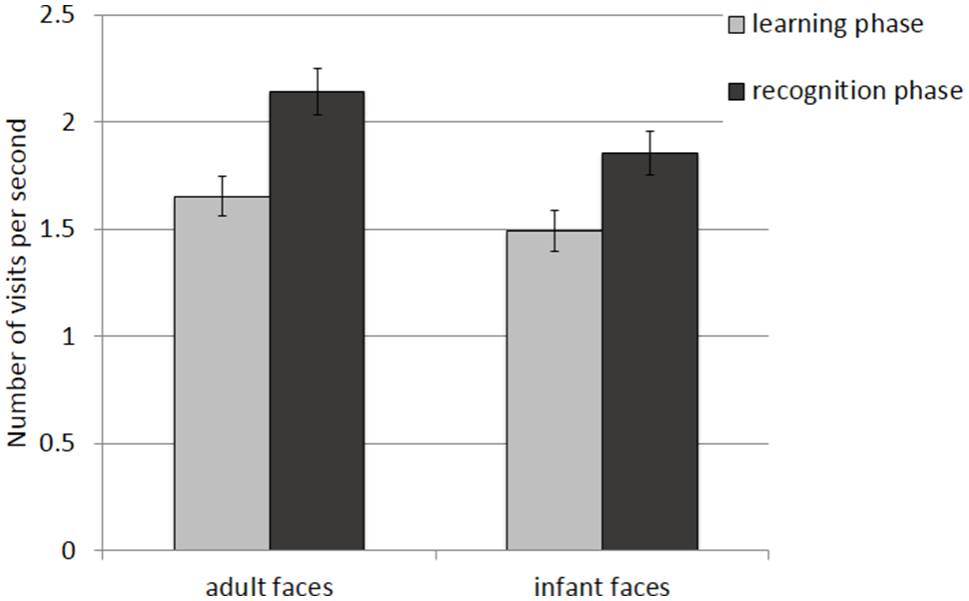
**Number of visits per second recorded on correct trials during the learning and recognition phase.** Error bars represent the standard error of the means.

The mean and SE of the number of visits for the adult and infant faces during learning and recognition are shown in **Table 3**. The 2 × 2 ANOVA with face age and phase as within-subjects factors showed main effects of face age, *F*(1,26) = 37.836, *p* < 0.001., η^2^ = 0.593, and phase, *F*(1,26) = 36.273, *p* < 0.001, η^2^ = 582, indicating that participants made more visits per second while encoding adult faces (*M* = 1.89, *SE* = 0.09) compared to infant faces (*M* = 1.67, *SE* = 0.09) and made more visits per second when recognizing faces (*M* = 1.99, *SE* = 0.10) than when learning faces (*M* = 1.57, *SE* = 0.09; see **Figure 6**).

#### Analyses on Incorrect Trials

##### Percentage of total viewing time

The mean and SE of the percentage of total viewing time on each AOI for the adult and infant faces during learning and recognition are shown in **Table 4**. The 2 × 2 × 3 ANOVA on the distribution of viewing time across the different AOIs for faces that were not correctly recognized during the recognition phase revealed a main effect of AOI, *F*(2,48) = 13.223, *p* < 0.001, η^2^ = 0.355, with shorter dwell time on the mouth region (*M* = 10.85%, *SE* = 1.95%) than on the eye (*M* = 31.14%, *SE* = 3.10%) and the nose region (*M* = 27.25%, *SE* = 2.746%). No other main effects or interactions attained significance, *p*s > 0.23.

##### Number of visits per second

The mean and SE of the number of visits for the adult and infant faces during learning and recognition are shown in **Table 3**. The 2 × 2 ANOVA with face age and phase as within-subjects factors revealed only a significant main effect of phase*, F*(1,26) = 16.854, *p* < 0.01, η^2^ = 0.413, suggesting that participants made more visits during the recognition phase (*M* = 1.87, *SE* = 0.10) compared to the learning phase (*M* = 1.48, *SE* = 0.10) and a main effect of face age, *F*(1,26) = 18.925, *p* < 0.01, η^2^ = 0.441, with more visits for adult faces (*M* = 1.79, *SE* = 0.09) compared to infant faces (*M* = 1.55, *SE* = 0.09).

#### Relations between Behavioral Performance and Eye Movements

To further explore the relation between scanning behavior and recognition performance we correlated percentage of viewing time on the eye and mouth region during the learning phase with two measures of behavioral performance – i.e., d’ and mean RTs – for adult and infant faces separately.

##### Percentage of total viewing time

Two-tailed Pearson correlation revealed that increasing percentage of dwell time on the eye region increased the likelihood of correct identification, as measured by d’, for infant faces *r* = 0.423, *p* = 0.028 (especially during learning, *r* = 0.497, *p* = 0.008). The same correlation failed to reach significance for adult faces, *r* = 0.315, *p* = 0.109. Percentage of viewing time on the mouth region during recognition showed positive correlation with mean RTs for correct recognition responses for both infant, *r* = 0.449, *p* = 0.019, and adult faces, *r* = 0.376, *p* = 0.053.

##### Number of visits per second

For both adult and infant faces number of visits during recognition was positively correlated with recognition accuracy (d’) (adult faces: *r* = 0.395, *p* = 0.041; infant faces: *r* = 0.385, *p* = 0.047).

## Discussion

The current study explored the impact of face age on the visual processing strategies employed during encoding and recognition of face stimuli.

In the only previous study investigating how face age modulates behavior, Firestone et al. (2007) looked at how young and older adults’ visual exploration strategies and recognition performance differ for young and older adult face stimuli. Here, we wanted to extend this first work by analyzing young adults scanning behavior on young adult faces and on a more physically distant and less experienced, face category, namely infant faces.

Analysis of our participants’ response performance provides evidence for the presence of an own-age bias. Results from our study confirmed the presence of the expected markers for the own-age bias, with higher recognition accuracy (d’) for adult compared to infant faces and a trend toward mean RTs being faster for the former than the latter. Other studies using similar paradigms found a weaker or absent own-age bias (Firestone et al., 2007). In spite of the methodological differences between this study and previous studies (Kuefner et al., 2008; Macchi Cassia et al., 2009b) comparing adults’ performance in the processing of adult and infant faces, the current results suggest that, in the absence of consistent experience with other-age faces, young adults show an advantage in the recognition of own-age compared to other-age faces.

Most interestingly, our eye movements data provide novel evidence that adult and infant faces elicit different gaze patterns in non-experienced adults. Both our variables of choice associated with participants’ looking behavior (percentage of total viewing time on each AOI and number of visits per second) significantly differed for the two face categories considered, with adult faces being associated with higher number of visits per second and higher percentage of viewing time on the eye region independently of the task participants had to perform (to memorize or to recognize the face). The first variable (number of visits per second) is indicative of the dynamicity of visual exploration since we considered a visit to the area whenever a fixation was performed in any of the AOIs preceded by a saccade originating either from another AOI or from a region of the face not included in any specific AOI. Therefore, the higher number of visit per second found in the processing of adult faces can be considered as an index of more dynamic visual exploration of these faces compared to infant faces.

Regarding the percentage of viewing time, the general pattern of attention to facial features found was consistent with previous research (Henderson et al., 2005; Flowe, 2011; Nakabayashi et al., 2012; McDonnell et al., 2014) showing that participants fixate more the eyes than other regions of the face. In addition, as predicted, in our data there were differences in how participants processed own-age vs. other-age faces. To this regard, our finding of a higher percentage of viewing time on the eye region of adult compared to infant faces seems to be at odds with what found in the study by Firestone et al. (2007) where young adults looked longer at the eye region of old faces compared to young faces. There are at least two important methodological differences between the current study and the Firestone et al.’s (2007) study that may explain the conflicting results. First of all, in Firestone et al.’s (2007) study participants’ eye movements were recorded during an age judgment task; the longer fixation time on the eye region of older adult faces compared to young adult faces might be explained as a consequence of the specific task demands. Participants had to focus on the age of the faces, and it is conceivable that they would have fixated the region that is more informative in that context, which is probably the eye given the presence of wrinkles. Secondly, participants in Firestone et al.’s (2007) study were not controlled for the amount of experience with older adult individuals and, as shown in previous studies, amount of contact can make an important difference in modulating perceptual strategies during the processing of older adult faces (Proietti et al., 2013). Additional evidence would be important to clarify if the inconsistency between our results and those obtained by Firestone et al. (2007) is due to a real effect of older adult faces as a peculiar face category or to the effect of task demands (i.e., age judgments compared to recognition task).

Nonetheless, it is important to underline that the results we obtained (higher percentage of viewing time on the eye region for own-compared to other-age faces) are in line with findings from studies on the race bias, showing that Caucasian participants dwell longer on the eye region of own-race faces compared to other-race faces (Goldinger et al., 2009; Wu et al., 2012; McDonnell et al., 2014, but see Blais et al., 2008; Caldara et al., 2010; Hills and Pake, 2013 for no differences in looking beahaviour for own-race vs. other-race faces). Previous studies have shown that adult participants rely on different perceptual strategies when processing own- and other-age faces by looking at phenomena such as the face inversion effect (e.g., Kuefner et al., 2008) or the composite effect (Kuefner et al., 2010). The present findings add to this earlier evidence by showing that part—though probably not all—of the difference in how individuals encode different categories of faces, being the differences related to age or race, lies in their differential attention to discrete facial features. At least in the case of Caucasian participants, the exploration of the eye region is an effective strategy more extensively employed in the processing of familiar face categories compared to unfamiliar face categories.

A second important finding from the current study relates to the difference in scanning strategies employed for encoding and recognition. In fact, the majority of existing studies on the age and race biases, analyzed participants’ looking behavior during face learning (Firestone et al., 2007; Goldinger et al., 2009). Our results suggest that scanning strategies change as a function of the task participants have to perform (encoding or recognizing a face). Specifically, results showed that regardless of face age, participants tended to focus their attention more on the eye region in the learning phase, while they tended to use a less specific strategy in the recognition phase. These findings seem to be at odds with those of an earlier study by Henderson et al. (2005) that showed that the distribution of looking time across face features becomes more restricted from learning to recognition, with increasing dwell time on the eye and nose regions and decreasing looking time to the other features (e.g., mouth, chin, forehead). However, there are many methodological differences that may explain inconsistency in the results. For example, each participant in Henderson et al.’s (2005) study was tested in two different learning conditions, only one of which was a free viewing condition as in our study. In the second condition participants had to keep their gaze steady in the area directly between the eyes. It is possible that this restricted viewing condition during learning has biased participants to keep their gaze within the same region even during recognition, thus restricting the distribution of fixations across face features. In addition, in the learning phase of the Henderson et al.’s (2005) study each face was presented for 10 s, whereas in the current study we used much shorter presentation duration (i.e., 3 s). It is possible that such a shorter presentation duration induced participants to focus their attention more on the most informative facial features (i.e., eyes), rather than moving attention across features. In any case, our findings do concord with those by Henderson et al. (2005) in pointing to the dominance of the eyes as an important (based on our data, the most important) feature for face learning.

In addition to that, our data also indicate that participants used a more dynamic strategy during recognition compared to learning, which is reasonable if we consider that participants have to explore all features in order to find the familiar cues coded during learning. Even more important, the use of a more dynamic strategy (higher number of visits) is functional to recognition, as indicated by the positive correlation between number of visits and recognition accuracy (d’). This finding suggests that, during the short time (*M* = 1735 ms) before the participant makes a recognition decision and provides his/her response, a global and more dynamic scanning of the whole face is more functional than a more analytic exploration of the features, for both adult and infant faces.

This conclusion is further supported by the finding that the amount of sampling of the eye region during learning in our data was, to some extent, associated with differences in recognition performance. The analyses conducted separately for correct and incorrect trials confirmed that the larger sampling of the eye region compared to the other AOIs for adult faces with respect to infant faces occurred only for those faces that were subsequently correctly recognized. This again suggests that the eyes are diagnostic to identity. This was confirmed by correlation analyses showing that viewing time on the eye region affected correct identity discrimination in the subsequent recognition phase. Of note, this was especially true for infant faces, whose eye region was viewed overall less than the eye region of adult faces; in the adult face condition, the overuse of the eye region may have masked the effect and led to the absence of a direct association between this exploration strategy and recognition accuracy. Therefore, correlation results combined with results from corrected vs. incorrect trials provide robust evidence of the relevance of the exploration of the eye region in sustaining efficient identity recognition.

Unlike the eye region, visual exploration of the mouth region resulted to be dysfunctional for subsequent face recognition as suggested by the fact that, for both adult and infant faces, longer inspection of the mouth is related to longer RTs in the identification of familiar faces. To the best of our knowledge, the only studies showing that visual exploration of the mouth region is important to face recognition are those using emotional faces (Eisenbarth and Alpers, 2011); in these cases it is clear that looking at the mouth represents an important strategy to gather information about the face. Since the faces used in the current study all posed a static, neutral expression, it is reasonable to assume that the mouth region didn’t provide any additional information diagnostic to identity recognition. Rather, dwelling on the mouth region at encoding led to longer RTs.

## Conclusion

This study provides novel evidence for the presence of an own-age bias in young adult individuals. This bias is evident not only in the recognition performance exhibited for adult compared to infant faces, but also in the employment of sampling strategies (longer looking at the eye region and a more dynamic exploration) during encoding, which are effective for accurate recognition. The selective use of these strategies for own-age faces was predominant during the encoding of novel faces more than during recognition of the familiarized faces, again pointing to the relevance of these strategies for efficient learning of facial identity.

The finding of differential scanning strategies for own-age as compared to other-age faces extends earlier evidence of perceptual processing differences between adult and infant faces in adults with limited experience with infants (Macchi Cassia et al., 2009a,b). The current study does not provide a direct test of the impact of experience on scanning patterns, as it lacks a comparison with experienced adults who have regular contact with infants. However, participants were intentionally selected for having very limited experience with infants according to the same inclusion criteria used in earlier studies that compared the magnitude of the OAB in novice and experienced participants (i.e., maternity-ward nurses, Macchi Cassia et al., 2009b; first-time mothers with younger siblings, Macchi Cassia et al., 2009a). In these studies (both cited in the Introduction) the experienced participants, unlike the novices, showed no (or smaller) sign of an OAB in perceptual recognition and a generalized inversion effect for adult and infant faces, suggesting that experience with infants is capable of modulating recognition ability and inducing the use of face-specific processing strategies in adulthood. In light of this earlier evidence, our finding that adult and infant faces elicit different gaze patterns in adults selected for having very limited experience with infants suggests that scanning strategies plays a critical role in the recognition advantage for faces belonging to the most familiar age categories.

## Conflict of Interest Statement

The authors declare that the research was conducted in the absence of any commercial or financial relationships that could be construed as a potential conflict of interest.
